# Choreoathetosis in the Setting of Human Herpesvirus-6 Infection in a Transplant Recipient

**DOI:** 10.5334/tohm.657

**Published:** 2021-10-13

**Authors:** Sarah Mancone, Chindhuri Selvadurai, Joachim Baehring, Amar Patel

**Affiliations:** 1 Yale School of Medicine, Department of Neurology, US

**Keywords:** Herpesvirus-6, Choreoathetosis, Chorea, Encephalitis, Leukemia

## Abstract

**Background:**

Human herpesvirus-6 (HHV-6) has been associated with various neurologic disorders, but movement disorders are rare. This case describes a patient who developed a choreoathetotic movement disorder in the setting of HHV-6 infection.

**Case Report:**

A 72-year-old woman with AML and recent HHV-6 encephalitis following stem cell transplant presented with involuntary movements. Neurologic examination demonstrated motor impersistence and irregular non-stereotyped writhing movements consistent with a choreoathetotic movement disorder secondary to HHV-6 infection.

**Discussion:**

This is the first literature reported case of adult-onset chorea associated with HHV-6 infection, though it remains unclear if the movement disorder was from the infection or a secondary autoimmune response.

## Introduction

Human herpesvirus-6 (HHV-6) has been associated with a broad spectrum of neurologic disorders in children and adults, but movement disorders in either group are rare with few published case reports in the literature. This case describes a patient who developed a choreoathetotic movement disorder in the setting of HHV-6 infection following allogeneic stem cell transplant for acute myeloid leukemia (AML).

## Case Description

A 72-year-old woman with a history of breast cancer in remission but complicated by secondary AML was admitted to the hospital for AML consolidation therapy. She was given cyclophosphamide, fludarabine, and total body irradiation followed by allogeneic stem cell transplant. Two and a half weeks post-transplant, she became acutely somnolent and febrile. An emergent computerized tomography (CT) of the head was obtained and unremarkable, magnetic resonance imaging (MRI) of the brain performed three days after symptom onset – though motion degraded – showed no acute abnormalities, and her initial infectious and metabolic work up was notable only for mild acute renal injury. A lumbar puncture showed 1 nucleated cell, 0 red blood cells, protein 20.3 mg/dL, and glucose 52 mg/dL with negative gram stain and bacterial culture. Despite the non-infectious appearing cerebrospinal fluid, she was found to have a positive cerebrospinal fluid HHV-6 quantitative polymerase chain reaction (PCR) that corresponded with rising HHV-6 viral titers in the serum – 12,419 copies/mL at onset of mental status change with a peak level of 105,862 three days later – raising concern for HHV-6 encephalitis. She was treated with intravenous ganciclovir followed by oral acyclovir prophylaxis with significant improvement in her mental status and a decrease in HHV6 viral load to <1,000 copies/mL five days following peak level.

A few weeks after her transplant she began to feel herself “shaking” both at rest and with activity. Over the next month she had worsening involuntary movements impairing her ability to feed herself leading to a referral to movement disorders clinic, and subsequent neurologic examination revealed mild intermittent dystonic head posture, motor impersistence, and continuous, irregular, non-stereotyped writhing movements of the head, neck, and upper limbs particularly involving hands and fingers symmetrically, consistent with choreoathetosis (see ***Video 1***). She had no history of exposure to neuroleptics, metoclopramide, or prochlorperazine, and there was no personal or family history of movement disorders. The patient was diagnosed with a choreoathetotic movement disorder secondary to HHV-6 infection, though it was unclear if the movement disorder was a sequela of the primary infection or a secondary autoimmune response. Differential diagnosis also included a choreoathetotic movement disorder due to mild renal failure and/or tacrolimus toxicity. These were deemed to be less likely causes, however, as her involuntary movements began subsequent to renal function improvement at a time when she had normal serum tacrolimus levels and no recent dosage changes. As she recovered from her HHV-6 infection, she had gradual symptom improvement. When seen one month after the movement disorder was initially observed she had less pronounced head movements and was able to cut food and eat by herself. She was treated with valbenazine 40 mg daily for one week during this period without additional improvement, so this was discontinued. When seen three months from the initial diagnosis of her movement disorder, she had only mild choreiform movements of the hands, worse on the left than the right, that were not bothersome to the patient. Three months after that, her symptoms had resolved and only recurred once in the setting of a brief febrile illness.

**Video 1:** **Video recording of patient’s neurologic examination.** This video shows the patient’s choreoathetotic movements at rest while answering questions testing her abstraction and memory.

## Discussion

Central nervous system involvement is a well-established risk of HHV-6 infection, and presentations can include meningoencephalitis, encephalopathy, epilepsy, demyelinating diseases, ataxia, opsoclonus-myoclonus, and cranial neuropathies [1]. In the case of reactivation after stem cell transplant, median onset of symptoms of HHV-6 encephalitis is approximately three weeks post-transplant [2]. Lack of pleocytosis and normal levels of protein and glucose are common and MRI typically demonstrates findings consistent with limbic encephalitis. Diagnosis is typically suggested by presence of a positive CSF HHV-6 PCR and appropriate clinical findings, and it can also be supported by elevated plasma HHV-6 PCR. In one study, it was shown that plasma HHV-6 DNA >10,000 copies/ml had 100% sensitivity and 64.6% specificity for HHV-6 encephalopathy. Movement disorders, specifically chorea, are very rare sequela of HHV-6 infection. Only two case reports in the literature describe children who developed chorea [1] or basal ganglia lesions [3] with primary HHV-6 infection, and only one case report describes an adult who developed a movement disorder, specifically parkinsonism, associated with HHV-6 reactivation [4] (see ***Table 1***). This is the first literature reported case of adult-onset chorea associated with HHV-6 infection. The rarity of this syndrome presented a unique diagnostic and therapeutic challenge, and it remains unclear if the movement disorder was secondary to the infection itself or an autoimmune response.

**Table 1:** Clinical features of movement disorders secondary to HHV-6 infection or reactivation.


	CASE 1 [1]	CASE 2 [3]	CASE 3 [4]

Age at onset	14 months	1 year	32 years

Sex	Female	Female	Male

Presenting symptoms	Fever and generalized clonic status epilepticus, hypotonia and developmental regression	Fever and exanthema subitum	Progressive parkinsonism, symmetric hyperreflexia and cognitive dysfunction 6 weeks after HSCT

Description of movement disorder	Progressive truncal ataxia, orofacial dyskinesias, and choreiform movements of the mouth, extremities, and trunk	Decreased voluntary movements and hand ataxia, unable to stand independently or maintain a sitting position	Bilateral bradykinesia, rest tremor, upper limb rigidity

CSF studies	11 WBCs (lymphocytic) Glucose 75 Protein 20 +HHV-6 A and B PCR	Normal -HHV-6 PCR (+HHV-6 IgM in serum)	45 WBCs (lymphocytic) Glucose 64 Protein 61 +HHV-6 PCR

MRI results	Bilateral periventricularand subcortical diffusion abnormalities related to post-ictal edema	Low intensity signals on T1-weighted images and high intensity signals on T2-weighted images in the bilateral putamen consistent with putaminal necrosis	Caudate and putamen hyperintensity in FLAIR and T2-weighted images and non-enhancing in Tl- weighted image

Treatment	Levetiracetam IVIg for 5 doses IV foscarnet for 2 weeks	No pharmacologic treatment	IV ganciclovir for 2 weeks Levodopa

Recovery	6 months after discharge patient remained seizure- free with resolution of chorea but persistent hypotonia, poor coordination. complete absence of expression language, other moderate developmental delays	1 year after discharge she had persistent mild dystonic or athetotic posturing of her extremities but was able to take several paces without support	Disorientation improved with ganciclovir and parkinsonism remained stable with a moderate response to levodopa


Abbreviations: HSCT, hematopoietic stem cell transplant; WBCs, white blood cells; HHV-6, human herpesvirus-6; PCR, polymerase chain reaction; MRI, magnetic resonance imaging; FLAIR, fluid-attenuated inversion recovery; IVIg, intravenous immunoglobulin; IV, intravenous.

For comparison, Sydenham chorea is a well-known post-infectious movement disorder, with the proposed mechanism being a “cross-reactive” autoimmune response against the brain induced by infection with streptococcus pyogenes [5]. While often self-limited, patients with intrusive persistent symptoms can improve with steroids and/or intravenous immunoglobulin (IVIg), and studies have shown better neuropsychiatric outcomes in patients who receive these treatments [5]. Choreiform movement disorders following HSV-1 encephalitis have also been observed and are thought to be secondary to post-viral autoimmunity [6]. In one review, 3 of 9 pediatric patients with relapsing chorea from HSV-1 infection were found to have elevated N-methyl-D-aspartate receptor antibodies and improved after early treatment with steroids, IVIg, and cyclophosphamide. The prototypic autoimmune encephalitis, anti-NMDAR encephalitis, is well established to be associated with movement disorders, including chorea and dystonia, with the clinical syndrome considered to be a likely immune response to the autoantibodies produced by this condition [5]. In a systematic review of the treatment of autoimmune encephalitis, it was shown that patients given early immune treatment did better than patients given no treatment or late treatment [5].

In our case, treatment of the infection with resolution of HHV-6 viral load in the serum, as well as time, led to symptom improvement and near-resolution over a period of 6 months. Though this patient had a positive outcome, it is unclear if speed of recovery could have been facilitated by early treatment with steroids or IVIg, as seen in conditions such as Sydenham chorea and anti-NMDAR encephalitis. If additional cases were to arise, further analysis of the serum and CSF could be performed to assess for autoantibodies suggestive of a secondary autoimmune mechanism as well as consideration of steroids or IVIg depending on the timeline and severity of symptoms to facilitate a faster and more complete recovery.
